# Refractive ocular conditions and reasons for spectacles renewal in a resource-limited economy

**DOI:** 10.1186/1471-2415-10-12

**Published:** 2010-05-07

**Authors:** Abdulkabir A Ayanniyi, Francisca N Folorunso, Feyisayo G Adepoju

**Affiliations:** 1 Department of Ophthalmology, College of Health Sciences, University of Abuja, Abuja, PMB 117, FCT, Nigeria; 2 Department of Ophthalmology, University Teaching Hospital, Ado Ekiti, Nigeria; 3 Department of Ophthalmology, University of Ilorin Teaching Hospital, IIorin, Nigeria

## Abstract

**Background:**

Although a leading cause of visual impairment and a treatable cause of blindness globally, the pattern of refractive errors in many populations is unknown. This study determined the pattern of refractive ocular conditions, reasons for spectacles renewal and the effect of correction on refractive errors in a resource-limited community.

**Methods:**

A retrospective review of case records of 1,413 consecutive patients seen in a private optometry practice, Nigeria between January 2006 and July 2007.

**Results:**

A total number of 1,216 (86.1%) patients comprising of (486, 40%) males and (730, 60%) females with a mean age of 41.02 years SD 14.19 were analyzed. The age distribution peaked at peri-adolescent and the middle age years. The main ocular complaints were spectacles loss and discomfort (412, 33.9%), blurred near vision (399, 32.8%) and asthenopia (255, 20.9%). The mean duration of ocular symptoms before consultation was 2.05 years SD 1.92. The most common refractive errors include presbyopia (431, 35.3%), hyperopic astigmatism (240, 19.7%) and presbyopia with hyperopia (276, 22.7%). Only (59, 4.9%) had myopia. Following correction, there were reductions in magnitudes of the blind (VA<3/60) and visually impaired (VA<6/18-3/60) patients by (18, 58.1%) and (89, 81.7%) respectively. The main reasons for renewal of spectacles were broken lenses/frame/scratched lenses/lenses' falling off (47, 63.4%).

**Conclusions:**

Adequate correction of refractive errors reduces visual impairment and avoidable blindness and to achieve optimal control of refractive errors in the community, services should be targeted at individuals in the peri-adolescent and the middle age years.

## Background

Worldwide refractive error is the cause of blindness in 8 million persons (18% of all causes of blindness second only to cataract) and the cause of visual impairment in 145 million persons accounting for over 50% of all causes of visual impairment [1,2].

Regardless of its contribution to visual impairment and treatable blindness globally, the pattern of refractive error in many populations is unknown more especially in resource-limited community such as Nigeria. However, some studies on refractive errors have been conducted in developed [3,4] and developing economies. For instance, in a population-based study in India [5], the prevalence of myopia was 3.19% and 19.45% among individuals who were fifteen years or less and above fifteen years old respectively. In the same study, hyperopia had prevalence of 62.62% and 8.38% in the respective aforesaid age groups. The study found refractive errors as significant eye condition in the population [5].

In Nigeria many studies on refractive errors were school-based [6,7] and only give information on the subset of population. On the other hand, population-based studies are expensive and time consuming but give accurate and representative data. However, an hospital-based study provides an easier and cheaper source of information that can be inferred on general population. A number of earlier hospital-based studies [8-10] elsewhere in Nigeria confirmed refractive errors as common in Nigeria. These studies were concerned with pattern of refractive errors. In one of such studies involving 1,824 patients, myopia, hypermetropia, and astigmatism were found in 39.2%, 23.33%, and 21.80% eyes respectively. The presbyopic error was diagnosed in 1,640 eyes [8].

The patients with refractive errors are frequent callers at various eye clinics, satisfaction in care and glasses prescribed will boost confidence in eye care services and increase yield in detection of other ophthalmic diseases. Some patients however due to lack of satisfaction from recently obtained glasses make a return visit in a short while. Various reasons why a client may be dissatisfied by a pair of glasses prescribed had looked at by some studies [11,12].

The patients with refractive errors are often cared for by a complement of ophthalmic team including ophthalmologists and optometrists working together to deliver eye care services. However, practices involving only individual eye care specialist abound, such a practice being run solely by an optometrist was reviewed in this paper.

It has been recommended in Nigeria that people above 40 years of age should have yearly routine eyes examination. However, it is rarely observed as most eye care services are located in the cities where about 20% of the population reside, making the services unavailable to the needy majority. It is pertinent to note that Nigeria is a resource-limited country, the Gross Domestic Product per capital is USD1,128 and 60% of Nigerians are below poverty line, earning less than USD1 per day [13]. Eye health care service in Nigeria is essentially users' self-financed. The National Health Insurance Scheme (NHIS) is relatively young and limited in operation, serving only individuals in organized governmental and non-governmental agencies.

The aims of this study were to provide information on the pattern of refractive ocular conditions, the reasons for spectacles renewal and document the effect of correction on refractive errors in a resource-limited community. It is believed the findings would assist the global effort at reducing burden of visual impairment/blindness caused by refractive errors.

## Methods

This was a retrospective analysis of the case records of all patients who came for eye consultations in a private optometry practice, Ado Ekiti, Nigeria between January 2006 and July 2007.

The information extracted from each patient's case record was entered into proforma. This included age, sex, presenting ocular complaints and duration of onset of symptoms, unaided and corrected visual acuities, the objective and subjective refractive powers and diagnoses. Further information on reasons for spectacles renewal and the duration of spectacles wear before consultations among patients already wearing spectacles were also noted.

The reasons for spectacles renewal as used in this study referred to spectacle's or patient's or prescriber's related factors as documented in the case records that led to 'sub-optimal' or unsatisfactory correction of refractive errors. Asthenopia referred to complaint of ocular discomfort, brow ache, photophobia, headache on prolonged use of eyes that got relieved with the correction of refractive errors. Where there was more than one complaint, the main/principal complaint was used in the analysis.

The refractive errors were diagnosed based on correction with at least +0.50 or -0.5 dioptre sphere (DS) or dioptre cylinder (DC) or combination. The diagnosis of myopia and hyperopia were based on refractive errors of at least -0.5 DS and +0.5DS respectively. The presbyopic patients had unaided near point range of 0.75 m to 1.25 m. The presbyopic error was diagnosed based on improvement in near vision range to 0.3 m to 0.4 m following correction with at least +0.75 DS.

Myopic astigmatism and hyperopic astigmatism were diagnosed based on refractive errors of at least -0.5 DC and +0.5 DC respectively. The diagnoses of co-existing refractive errors in a single eye also are included in this report due to relative subjective improvement in vision. For instance, presbyopia with hyperopia referred to coexisting presbyopic and hyperopic refractive errors in the same eye. The presbyopia with myopia is coexisting presbyopic and myopic refractive errors in the same eye.

One hundred and ninety-seven patients whose case records had incomplete information and those whose complaints/diagnoses were not related to refractive ocular conditions were excluded from the analysis. The age and gender patterns of the excluded patients were similar to those analysed.

This study was carried out following the guidelines as contained in the declaration of Helsinki. The Ethical approval to carry out this study was obtained from the Ethical Review Committee of Folnex Optometry practice, Ado Ekiti, Nigeria.

The extracted information was entered into SPSS 15 and analysed.

## Results

### Patients demographic characteristics

**Figure 1:**
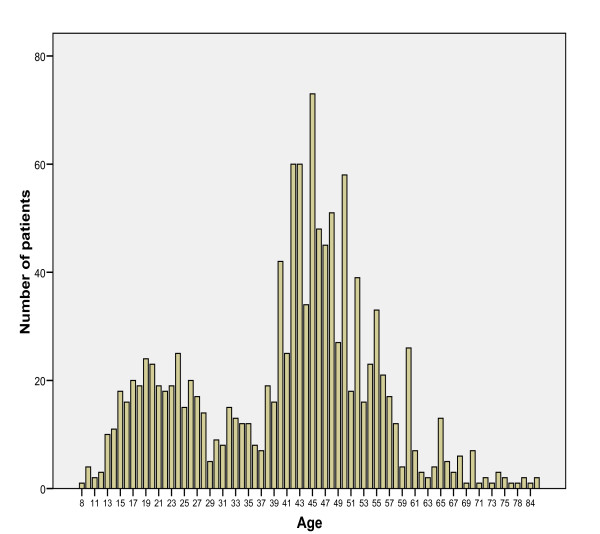
**Age distribution of the patients**.

A total number of 1,216 out of 1,413 patients (86.1%) who were seen at the Folnex Optometry Practice, Nigeria, within the study period were included in the analysis. They comprised of 486 (40%) males and 730 (60%) females (M: F = 1:1.5) with age range 8 to 85 years, mean age 41.02 years SD 14.19. The age distribution of these patients that sought optometry care peaked at peri-adolescent and the middle age years (Figure 1). Most patients were between the second and sixth decades of life with a peak at fifth decade (median age 44.00, modal age 45) (Figure 1).

### Refractive ocular conditions

**Figure 2:**
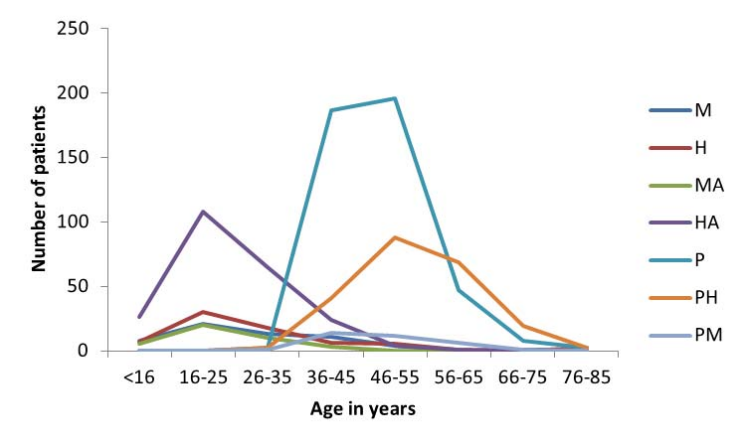
**Age distribution of refractive errors**. M = myopia, H = hyperopia, MA = myopic astigmatism, HA = hyperopic astigmatism, P = presbyopia, PH = presbyopia with hyperopia, PM = presbyopia with myopia

The numbers of patients with myopia and myopic astigmatism were less than patients with hyperopia and hyperopic astigmatism respectively. The aforesaid four refractive errors peaked at 16-25 age group while presbyopia peaked at 45-55 age group (Figure 2).

Many patients visited optometry clinic on account of complaints bordering mainly on spectacles, blurred near vision and asthenopia (Table 1). The presbyopia was the commonest refractive error among others (Table 1). The range of onset of ocular symptoms among 140 patients (11.5%) before seeking optometrist assistance was 0.04 - 10.00 years, mean 2.05 years SD 1.92 (1 year each for the median and the mode). The range of use of spectacle (among patients who were already using spectacles) before optometry consultations was 0.1 - 25 years, mean 3.58 years SD 3.10, mode 2 years.

**Table 1:** Presenting complaints and refractive conditions among the patients.

Complaints and refractive conditions			Male	Female	Total (%)
**Principal Complaints (n = 1216)**					
Blurred near vision			174	225	399 (32.8)
Blurred distant vision			32	42	74 (6.1)
Blurred near and distant vision			30	46	76 (6.3)
Spectacle related (lost glasses, glasses discomfort)			177	235	412 (33.9)
Ocular discomfort: pains, photophobia etc			73	182	255 (20.9)
**Refractive conditions (n = 1216)**					
Presbyopia			199	232	431 (35.4)
Presbyopia with hyperopia/hyperopic astigmatism			115	161	276 (22.7)
Presbyopia with myopia/myopic astigmatism			30	34	64 (5.3)
Presbyopia with astigmatism			4	8	12 (1.0)
Hyperopia			20	49	69 (5.7)
Hyperopic astigmatism			71	169	240 (19.7)
Myopia			24	35	59 (4.9)
Myopic astigmatism			20	40	60 (4.9)
Others			3	2	5 (0.4)

Generally, there was improvement in aided visual acuity following correction (Table 2). More importantly, there were reductions in the magnitudes of the blind and visually impaired patients (Table 3).

**Table 2:** Distribution of unaided and corrected visual acuity by eyes, (n = 1,216).

VA	VARE		VALE	
	
	Unaided (%)	Corrected (%)	Unaided (%)	Corrected (%)
≥6/18	1076 (88.5)	1187 (97.6)	1088 (89.4)	1189 (97.8)
<6/18-6/60	108 (8.9)	15 (1.2)	101 (8.3)	18 (1.5)
<6/60-3/60	1 (0.1)	1 (0.1)	2 (0.2)	1 (0.1)
<3/60-PL	31 (2.5)	13 (1.1)	25 (2.1)	8 (0.6)

VA = visual acuity, VARE = visual acuity right eye, VALE = visual acuity left eye, PL = perception of light

**Table 3:** Percentage reduction in visual impairment/blindness by eyes/patients.

VA	Unaided (%)		Corrected (%)		Reduction (unaided less corrected)	
	
	Eyes	Patients	Eyes	Patients	Eyes (%)	Patients (%)
<6/18-3/60	212	109	35	20	177 (83.5)	89 (81.7)
<3/60-PL	56	31	21	13	35 (62.5)	18 (58.1)

VA = visual acuity, PL = perception of light

### Reasons for spectacles renewal

The reasons why patients sought renewal of their spectacles due to 'sub-optimal' or unsatisfactory correction of refractive errors were mostly spectacles related among others (Table 4).

**Table 4:** Reasons for renewal of spectacles at the optometrist clinic.

Reasons	No (%)
**Spectacle related**	
Broken lenses/frame/scratch lenses/lens falling	47 (63.4)
**Patient related**	
Aphakia	5 (6.7)
Oculocutaneous albinism	4 (5.3)
Pterygium	2 (2.6)
Non appealing frame	2 (2.6)
Malingering	2 (2.6)
*Others	11 (15.4)
**Prescriber related**	
Incorrect prescription	1 (1.4)

*Others: 1 (1.4%) each case of cataract, cornea scar, glaucoma, diabetic mellitus, photophobia, macular scar, hypertension, ocular trauma, pathological myopia, uniocular blindness, spectacle purchased in the open market

## Discussion

The gender pattern in this study showed females were one and half times more than their males' counterparts even with excluded group taken into consideration. Refractive errors are reported to be commoner among females and females seek optometry consultations than males [11,14].

The two peaks of increased number of patients that sought optometry consultations are remarkable. The first peak which coincides with teenage years up to mid-twenty of age is a period of changing ocular refractive state where correction would be desired by the patients. It coincides with the growing and developmental stage of life [15], a period of schooling where normal visual acuity would be required. The second peak coincides with middle age. This is a period where presbyopic correction is most required in view of diminishing accommodation [16,17].

It is of interest the magnitude of patients with myopia appeared reduced compared to hyperopia especially in the 16-25 age group. In contrast to a similar but earlier hospital-based study in Nigeria, myopia was the commonest refractive error in the study population [11]. The number of myopic patients is expected to be more in the 16-25 age group. Myopia is responsible for much of the uncorrected refractive errors in the world [18]. In India, a population-based study showed among ≤15 years old, myopia increased with increasing age, and hyperopia prevalence was greater among <10 years old. However, among >15 years old, myopia and hyperopia increased with increasing age [8]. The observed pattern of the refractive errors in this study has raised issues. Could it be, more patients with hyperopic errors presented in the optometry clinic than their myopic counterparts or hyperopic error is more prevalent in the community where the optometry clinic is located? On the other hand, the magnitude of the patients with astigmatic errors was more than patients with spherical errors in this study. As elsewhere [8,19], there is need to conduct population-based study to determine the pattern of refractive errors in this community.

The number of patients with presbyopic symptoms agreed with the number diagnosed of presbyopia. The study found presbyopic symptoms among the most common ocular complaints by the patients and presbyopia as the most common diagnosis. This was not unexpected as over two-thirds of the patients were in the presbyopic age range. The preponderance of patients in the presbyopic age group relative to the other age groups might be borne out of the fact that they were in the working class of the society, and most of them should be able to self-finance optometry care better than other age groups where, most might be dependants. On the other hand, presbyopia being the commonest refractive error in this report might be related to hot tropical climate of the study community, presbyopia is reported to have earlier onset especially among females in hot climates [20].

This study found that on average the patients sought optometrist care two years after the onset of symptoms bordering on refractive ocular conditions. However, the median and the modal ages at which the patients sought optometrist care were equal, one year after the onset of symptoms. This signifies an important landmark, as beyond this period, most patients may not be able to cope with their visual activities without refractive correction. This no doubt, would be of predictive value and be of assistance during patient counselling sessions.

In this study, significant number of patients (26.6%) had optometric consultations on account of their old spectacles which were no longer comfortable. The average duration of the use of spectacles before the patients' wanted to change their spectacles due to a number of reasons including discomfort was three and half years while median duration for similar reason was three years. However, most patients opted for change at 2 years of use. In a population-based study in India, of those patients who had used spectacles previously, 43.8% discontinued based on feeling of incorrect prescription or the spectacle being uncomfortable [21].

This study showed a general improvement in aided visual acuities following correction. More importantly, there were reductions in magnitudes of the blind and the visually impaired patients following correction. This underscores the fact that the appropriate correction of refractive ocular conditions reduces the burden of visual impairment [3,5,22].

Also, this study found that the reasons for renewal of spectacles could be spectacles', patients' and prescribers' related. Spectacle borne factors including broken lenses, scratch lenses, lenses' falling off and broken frame can frustrate global efforts at adequate correction of refractive errors. The patients at the time of getting the spectacles should be educated [23] by dispensing opticians on the available lenses' choices and the proper handling of the spectacles to reduce the magnitude of this problem. On the other hand the proper fitting of the lenses by dispensing optician should reduce cases of lenses' falling off.

Some patients, (7.1%) made optometric consultations on account of their lost spectacles with consequential visual discomfort. In a population-based study of spectacles use in southern India, as many as 19.6% of people using spectacles had lost the pairs and could not afford to buy another pairs [21]. The patients should be counselled on how to take good care of their spectacles to reduce cases of lost spectacles. Through this, needless worries including ocular discomfort and cost of replacing lost spectacles would be avoided. Of course, replacing lost spectacles can be a luxury, regardless of lost quality of life, to many patients who cannot afford eye care services [24].

Note must; also, be taken of other reasons for spectacles renewal including ocular pathology, malingering and non-appealing frame, their fewer numbers notwithstanding, in view of their significance to adequate correction of refractive errors. A number of ocular pathologies including diabetes mellitus, hypertension, pterygium among others were found among the study population. Changes in the blood sugar levels in diabetes mellitus can induce 'myopic' or 'hyperopic' shift [25] (that disappears with optimal blood sugar control) and pterygium can induce refractive changes [26-28].

The eye care providers should rule out these diseases or ensure they are managed preferably before refraction and after. Spectacles prescribed without cognisance of these 'co-morbidities' would lead to sub-optimal correction of the refractive errors. Appropriate referrals by the optometrists [29] of diabetic and hypertensive patients to medical practitioners for management are very essential to optometric practice. Of course, this would prevent the prescription of needless spectacles that would not benefit patients. Such patients can be appropriately refracted at a later date if necessary.

This study is limited by its retrospective nature and the findings should be interpreted with caution. There are information that could enrich this study but for its retrospective nature. For instance, the number of patients that actually picked corrective pair of spectacles as well as the number that failed to do so and why. It is very important that optometry clinics follow a 'standard protocol' for documenting patients' information, regardless of their location and tight schedule in order to bridge the important missing gaps. Moreover, a population-based study on refractive errors among the people of the study area as earlier suggested would complement this study. Nevertheless, this study is representative of the features of refractive ocular conditions in an optometry practice in a resource-limited economy.

## Conclusions

Adequate correction of refractive errors reduces visual impairment and avoidable blindness and to achieve optimal control of refractive errors in the community, services should be targeted at individuals in the peri-adolescent and the middle age years. The knowledge of the mean duration of refractive errors symptoms would be useful in patients counselling. The knowledge of the pattern of refractive ocular conditions and reasons for spectacles renewal in a resource-limited community would aid global efforts at controlling avoidable blindness/visual impairment.

## Competing interests

The authors declare that they have no competing interests.

## Authors' contributions

AAA: Conceptualization and study design; Data Analysis; Drafting and revising manuscript. FNF: Acquisition of data; Reading and revising manuscript. FGA: Contributed to study design, Reading and revising manuscript. All the three authors approved the manuscript to be published.

## Pre-publication history

The pre-publication history for this paper can be accessed here:

http://www.biomedcentral.com/1471-2415/10/12/prepub
